# Individualised stepwise adaptive treatment for 3–6-year-old preschool children impaired by attention-deficit/hyperactivity disorder (ESCApreschool): study protocol of an adaptive intervention study including two randomised controlled trials within the consortium ESCAlife

**DOI:** 10.1186/s13063-019-3872-8

**Published:** 2020-01-09

**Authors:** Katja Becker, Tobias Banaschewski, Daniel Brandeis, Christina Dose, Christopher Hautmann, Martin Holtmann, Thomas Jans, Lea Jendreizik, Carolin Jenkner, Katja John, Johanna Ketter, Sabina Millenet, Ursula Pauli-Pott, Tobias Renner, Marcel Romanos, Anne-Katrin Treier, Elena von Wirth, Anne-Kathrin Wermter, Manfred Döpfner

**Affiliations:** 1 Department of Child and Adolescent Psychiatry, Psychosomatics and Psychotherapy, Medical Faculty of the Philipps-University Marburg, Hans-Sachs-Str. 6, 35039 Marburg, Germany; 2 Center for Mind, Brain and Behavior (CMBB), University of Marburg and Justus Liebig University Giessen, Marburg, Germany; 3 Department of Child and Adolescent Psychiatry and Psychotherapy, Central Institute of Mental Health, Heidelberg University, Medical Faculty Mannheim, Mannheim, Germany; 4 Department of Child and Adolescent Psychiatry, Psychosomatics and Psychotherapy, Medical Faculty of the University of Cologne, Cologne, Germany; 5 School of Child and Adolescent Cognitive Behaviour Therapy (AKiP), University Hospital of Cologne, Cologne, Germany; 6 Department of Child and Adolescent Psychiatry and Psychotherapy, LWL-University Hospital Hamm, Ruhr-University Bochum, Hamm, Germany; 7 Centre of Mental Health, Department of Child and Adolescent Psychiatry, Psychosomatics and Psychotherapy, University Hospital of Würzburg, Würzburg, Germany; 8 Clinical Trials Unit Freiburg, Medical Centre – University of Freiburg, Faculty of Medicine, University of Freiburg, Freiburg, Germany; 9 Department of Child and Adolescent Psychiatry and Psychotherapy, University Hospital Tübingen, Tübingen, Germany

**Keywords:** Attention-deficit/hyperactivity disorder, ADHD, Preschool children, Stepped care, Adaptive treatment, Telephone-assisted self-help, Behaviour therapy, Kindergarten, ESCAlife

## Abstract

**Background:**

Attention-deficit/hyperactivity disorder (ADHD) is a psychosocially impairing and cost-intensive mental disorder, with first symptoms occurring in early childhood. It can usually be diagnosed reliably at preschool age. Early detection of children with ADHD symptoms and an early, age-appropriate treatment are needed in order to reduce symptoms, prevent secondary problems and enable a better school start. Despite existing ADHD treatment research and guideline recommendations for the treatment of ADHD in preschool children, there is still a need to optimise individualised treatment strategies in order to improve outcomes. Therefore, the ESCApreschool study (Evidence-Based, Stepped Care of ADHD in Preschool Children aged 3 years and 0 months to 6 years and 11 months of age (3;0 to 6;11 years) addresses the treatment of 3–6-year-old preschool children with elevated ADHD symptoms within a large multicentre trial. The study aims to investigate the efficacy of an individualised stepwise-intensifying treatment programme.

**Methods:**

The target sample size of ESCApreschool is 200 children (boys and girls) aged 3;0 to 6;11 years with an ADHD diagnosis according to Diagnostic and Statistical Manual of Mental Disorders, Fifth Edition (DSM-5) or a diagnosis of oppositional defiant disorder (ODD) plus additional substantial ADHD symptoms. The first step of the adaptive, stepped care design used in ESCApreschool consists of a telephone-assisted self-help (TASH) intervention for parents. Participants are randomised to either the TASH group or a waiting control group. The treatment in step 2 depends on the outcome of step 1: TASH responders without significant residual ADHD/ODD symptoms receive booster sessions of TASH. Partial or non-responders of step 1 are randomised again to either parent management and preschool teacher training or treatment as usual.

**Discussion:**

The ESCApreschool trial aims to improve knowledge about individualised treatment strategies for preschool children with ADHD following an adaptive stepped care approach, and to provide a scientific basis for individualised medicine for preschool children with ADHD in routine clinical care.

**Trial registration:**

The trial was registered at the German Clinical Trials Register (DRKS) as a Current Controlled Trial under DRKS00008971 on 1 October 2015. This manuscript is based on protocol version 3 (14 October 2016).

## Background

Attention-deficit/hyperactivity disorder (ADHD) is a highly prevalent, early-onset, persistent neurodevelopmental disorder, which is associated with psychosocial functional impairment and a markedly reduced subjective health-related quality of life [1–3]. According to Diagnostic and Statistical Manual of Mental Disorders, Fifth Edition (DSM-5) or International Classification of Diseases, Tenth Edition (ICD-10) criteria, it is characterised by age-inappropriate, pervasive and persistent inattentiveness, impulsivity and/or motor restlessness [4, 5]. ADHD symptoms can be observed as early as the preschool years, with an estimated prevalence of 1.5–6% among preschool children [6, 7]. For the diagnosis of ADHD, clinically relevant functional impairment must be present in different settings, e.g. in the family and at preschool. In addition, comorbidity in preschool children with ADHD is common, with oppositional defiant disorder (ODD), communication disorder and anxiety disorders being the most prevalent comorbid conditions [8]. Early interventions have been shown to be particularly helpful and might prevent the development of secondary symptoms as well as school failure [9–11]. International and national treatment guidelines [12–14] recommend a combination of multiple, individually adapted treatment components (i.e. multimodal therapy). However, compared with school-age children, treatment with immediate-release methylphenidate has been shown to be less effective in preschool children (i.e. effect sizes were considerably smaller), to cause more adverse events and to be less accepted by parents [15]. In contrast, psychosocial treatment may be most powerful at this early age, as it can positively influence parental scaffolding during early self-regulation development and prevent the development of coercive cycles of negative parent-child interactions. Accordingly, clinical guidelines recommend psychosocial interventions in the family and the preschool as the treatment of choice for preschool children with ADHD [12–14].

Parent counselling and parent management training have been found to be effective treatments for children of this age group [12, 16, 17]. Recent meta-analyses on the efficacy and effectiveness of psychosocial interventions in preschool children with disruptive behaviour disorders (DBD), including ADHD, showed medium to large effects on child behaviour outcomes. Based on 13 studies, Charach et al. [18] found a moderate overall effect (standardised mean difference [SMD] = 0.75) of parenting training on parent-reported DBD symptoms; the effect size for core symptoms of ADHD was SMD = 0.77 (five included studies). Similarly, a meta-analysis of 36 randomised control trials (RCTs) on a broader range of psychosocial interventions resulted in a large overall effect on parent-, teacher- and observer-reported DBD symptoms (Hedges’ *g* = 0.82) and a medium effect (*g* = 0.61) on hyperactivity/impulsivity symptoms in particular [19]. The analysis included behavioural and non-behavioural treatments, with the former showing significantly larger effects [19] . Even though clinical guidelines recommend preschool interventions, so far, preschool teacher training as well as preschool-based interventions are rare. The Cologne group led by Döpfner reported that their indicated prevention programme addressing preschool teachers was effective [9, 20, 21]. The measured effects were largely maintained at 1-year follow-up (e.g. [9, 22]).

However, previous studies in preschool children with ADHD are subject to some limitations. For instance, the evidence regarding the value of these interventions is limited to unblinded ratings made by individuals who are likely to be invested in the treatment success. Evidence of efficacy from well-controlled trials using blinded assessments of outcomes is still lacking [23]. Furthermore, the validity of the available RCTs is limited by the design characteristics, as most of the RCTs used no treatment as a control condition, rather than treatment as usual (TAU) or non-specific support. Therefore, some of these results cannot be generalised [18, 19].

Another critical issue in the treatment of preschool children with ADHD is that unfortunately, not all parents are willing or able to procure treatment for their children. Frequent reasons why parents do not start or complete interventions include a lack of problem awareness, a lack of availability of psychotherapy, or other problems such as transport, childcare, work commitments, financial burden, or stigma (e.g. [24, 25]). Besides and despite these treatment barriers, the need for intervention still exceeds the number of available treatment options, and treatment resources are sparse [24]. Therefore, it is important to focus on therapies and dissemination methods that help to overcome these barriers. There is evidence that self-directed, bibliographic interventions and telephone- or web-based assistance might be one way forward [18, 26, 27]. Self-directed interventions have been shown to be effective in reducing parent-rated externalising behaviour problems [28, 29]. Some studies indicate that the effects of such interventions may be improved by minimal therapeutic support (e.g. by telephone; see [28]). For example, Kierfeld et al. [30] successfully demonstrated the effects of a telephone-assisted self-help (TASH) intervention for parents of preschool children with ADHD and other externalising behaviour problems. The effects were maintained at 1-year follow-up [31]. An effectiveness study on a TASH intervention for parents of 6–12-year-old children with ADHD found a significant reduction of ADHD and comorbid symptoms under routine care conditions [32]. Moreover, TASH for parents was found to enhance effects of methylphenidate treatment in a sample of children with ADHD [33]. Interestingly, behavioural and non-behavioural TASH interventions seem to have similar effects [34].

An open research question is which of the different treatment components (e.g. behaviour therapy, self-directed interventions) should be offered following obligatory psychoeducation, and in which order. In this respect, a stepped care approach in which treatment is individually adapted according to symptom strength, comorbid symptoms, specific family needs and treatment response is suggested [12]. However, empirical evidence for the efficacy of adaptive treatment strategies for patients with an ADHD diagnosis in general, and in preschool children in particular, is sparse. A study assessing an adaptive multimodal treatment in school-age children found that both behaviour therapy and a combination of behaviour therapy and pharmacotherapy are effective in the treatment of ADHD [35], with effects persisting at an 18-month follow-up [36]. Whereas the efficacy of different singular interventions is also well documented in preschool children, a stepwise approach with individualised adaptive treatment strategies has not been empirically validated for this age group. Therefore, a stepped care approach for 3–6-year-old preschool children is being evaluated within the trial ESCApreschool (Evidence-Based, Stepped Care of ADHD in Preschool Children aged 3 years and 0 months to 6 years and 11 months of age (3;0 to 6;11 years). The results aim to improve the knowledge about individualised treatment strategies for preschool children with ADHD. The evaluation of a stepwise approach in routine care is of particular importance for clinical practice.

## Methods and Design

ESCApreschool is part of a multicentre consortium studying stepped care approaches for the treatment of ADHD along the lifespan (ESCAlife: Evidence-Based Stepped Care of ADHD along the Lifespan, coordinator Tobias Banaschewski). ESCAlife encompasses stepped care designs in different age groups (preschool age, school age, adolescents, adults), each focusing on the different specific needs in the respective life phases, including 6–12-year-old school children (ESCAschool [37]), 12–17-year-old adolescents (ESCAadol [38]) and 16–45-year-old adults (ESCAlate [39]). With regard to design and methodology, the single studies overlap to allow for the examination of selected research questions across all age groups.

### Objectives, study design and trial flow

ESCApreschool aims to examine the efficacy of an individualised stepwise-intensifying treatment approach based on evidence-based behavioural interventions in patients with ADHD or patients with ODD and additional ADHD symptoms, aged 3;0 to 6;11 years, who attend preschool. Different treatment strategies are investigated for children who respond to a low-threshold TASH intervention and those who do not. A further question is to determine precisely which families benefit from the low-threshold TASH intervention or more intensive behaviour therapy and which families do not, and to identify the predictors and moderators of treatment response. Therefore, the secondary objective is to examine the predictability of treatment response by psychological and biological variables.

The multicentre study is designed as a stepwise (two steps) adaptive treatment study including two RCTs. In the adaptive design, the second step of the trial (step 2) depends on the outcome of step 1.

Step 1 of the ESCApreschool study consists of a randomised waitlist-controlled trial which provides the parents (and optionally also the preschool teachers) of the participating children (planned number of *N* = 200 children) with a 3-month TASH intervention. The parents (and preschool teachers) are randomised to receive this treatment either immediately at the beginning of the trial or after a 3-month waiting period.

**Fig. 1:**
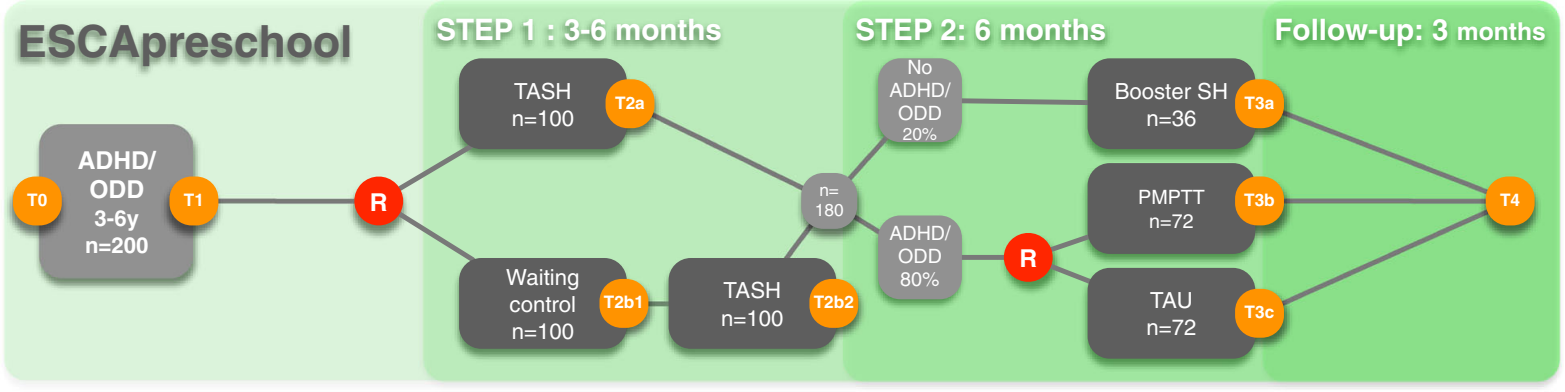
Flow chart. ADHD = Attention-Deficit/Hyperactivity Disorder; ODD = Oppositional Defiant Disorder, T0 to T4 = Assessment Time Points; R = Randomisation; TASH = Telephone-Assisted Self-Help for Parents and Preschool Teachers; PMPTT = Parent Management and Preschool Teacher Training, TAU = Treatment as Usual, Booster SH = Booster Self-Help

The intervention provided in step 2 of the trial depends on the outcome of the low-threshold TASH intervention. If children fully respond to this intervention, their parents (and preschool teachers) receive TASH booster sessions in step 2. If children do not or only partially respond to TASH, that is, if they show persisting ADHD and/or ODD symptoms, they are randomised to receive either parent management and preschool teacher training (PMPTT) or TAU. For an overview of the trial flow, please refer to Fig. 1.

The sample sizes and response rates displayed in the figure are estimations and therefore differ from the actual recruitment and response rates.

Step 1 lasts for 3–6 months depending on the allocation of the participants (3 months in the TASH group; 6 months in the waiting control group, which undergoes a 3-month waiting period followed by the 3-month TASH intervention period). Step 2 lasts for 6 months, and the follow-up period lasts for 3 months.

Measurements are taken at T0 and T1 (T0 = screening of inclusion and exclusion criteria and assessment of ADHD and ODD criteria; T1 = baseline assessment as described below). Further measurements ensue after step 1 (T2), after the second treatment phase in step 2 (T3), and 3 months after the end of the treatment (follow-up examination; T4). Families who are randomised to the waiting control group take part in an additional assessment after the waiting period (T2b1). Participants who discontinue the intervention during one of the treatment phases are also invited for the follow-up assessment (T4) in order to monitor their development. Additionally, data are collected during the therapeutic process.

Clinical assessments of ADHD and ODD symptom severity are completed by trained experienced clinicians (further referred to as “blinded clinicians”), who are blind to the patients’ assignment to treatment condition but not to the assessment time point (T1-T4). The inclusion of participants into the study, as well as their classification as full responders or partial/non-responders to step 1, are based on these clinical interviews. For validation purposes, all interviews are recorded and a subsample of the recordings is subsequently rated by a clinician who is blind to both the treatment condition and the assessment time point.

### Trial sites

At the start of the study, a total of six trial centres located at departments of child and adolescent psychiatry at university hospitals in Germany (Cologne, Hamm, Mannheim, Marburg, Tübingen, Würzburg) contributed to this multicentre trial.

The leading and coordinating centre of the ESCApreschool study is Marburg (Principal Investigator [PI] Katja Becker). Each of the six centres was expected to enrol between 30 and 40 patients in order to achieve a total sample size of *N* = 200 patients. To compensate for low recruiting numbers, three additional trial centres were included in 2018 (Aachen [Kerstin Konrad], Göttingen [Luise Poustka], Neuruppin [Michael Kölch]).

The TASH work group at the Cologne University Hospital (Manfred Döpfner, also Co-PI of ESCApreschool) is responsible for delivering TASH (which is provided centrally from Cologne for all participants). All other diagnostic procedures and treatments are provided at the respective study centres. On-site therapists who have been trained and who are supervised by the Cologne work group perform the behaviour therapy. Responsibility for data management, archiving and monitoring, as well as biometrics and project management, lies with the Centre for Clinical Studies in Freiburg.

### Participants

A total of 200 girls and boys, aged 3;0 to 6;11 years, with either a diagnosis of ADHD or substantial ADHD symptoms combined with a diagnosis of ODD are the study’s target group. Inclusion and exclusion criteria are shown in Table 1 (see Measures section for a more detailed description of the instruments). These data are assessed at baseline through an interview gathering baseline characteristics as well as sociodemographic data. Patients are recruited through the centres’ outpatient clinics, which are highly experienced in the treatment of ADHD or preschool children. Further recruitment strategies include the dissemination of information regarding the study at local conferences, and by contacting paediatricians, child and adolescent psychiatrists, child and adolescent psychologists and child guidance centres. Additionally, information is provided to other counselling centres, ADHD self-help groups and preschool teachers either in writing, in person, or through lectures. In Germany, the local health authorities organise a mandatory health examination for preschool children shortly before school entry. Therefore, the respective local health authorities are also informed about the study. All of these recruitment activities are flanked by homepage information, local public lectures and repeated advertisement actions providing information about the study (postings, flyers, bus advertisements, newspaper articles) in order to reach parents directly.

**Table 1:** Inclusion and exclusion criteria

Inclusion criteria	• Age 3;0 to 6;11 years* • Attending preschool* • Exhibiting externalising behaviour problems, that is, either meeting DSM-5 criteria for ADHD on a clinician-rated ADHD checklist (DCL-ADHS) or meeting DSM-5 criteria for ODD on a clinician-rated ODD-checklist (DCL-SSV) plus substantial attention or hyperactivity/impulsivity problems (score ≥ 0.7 on either the inattention or the hyperactivity/impulsivity subscale of the DCL-ADHS)* • Informed consent of the parents (and, if applicable, the preschool teacher*) and assent of the child
Exclusion criteria	• Intelligence quotient (IQ) < 80 • Comorbidity: pervasive developmental disorder, schizophrenia, bipolar disorder, severe depressive episode** • Lack of sufficient German-language skills of parents • Psychotropic medication of the child (except for ADHD medication)* • Child’s participation in a regular intensive behaviour therapy (on a weekly or fortnightly basis)

*Some changes have been made since the first version of the study protocol (9 June 2015). First, we initially planned an age span of 3;0 to 5;11 years. However, this resulted in the exclusion of 6-year-olds who were still attending preschool within the overall ESCAlife study. Thus, this inclusion criterion was changed to 3;0 to 6;11 years (Note to File G005 22 September 2016, positive Ethics Committee vote 08 November 2016). Second, the first version of the study protocol comprised an additional inclusion criterion of a time span from at least 9 months before enrolment in primary school. Due to recruitment problems, this criterion was changed to “attending preschool” (Note to File G003 25 May 2016; positive Ethics Committee vote 3 June 2016). Third, an inclusion criterion in the first version of the trial protocol was the presence of an ADHD diagnosis according to DSM-5, assessed with a clinician-rated ADHD checklist (DCL-ADHS). However, after study start, we realised that at preschool age, the differentiation between a diagnosis of ADHD and a diagnosis of ODD plus additional ADHD symptoms might be blurred. Therefore, this inclusion criterion was changed to that mentioned in the table (Note to file G002, 25 May 2016, positive Ethics Committee vote 3 June 2016).

Fourth, the initial inclusion criterion “informed consent of preschool teacher” was withdrawn due to the fact that in some centres, it was not permitted to contact preschool teachers directly. Therefore, in the present version of the study protocol, while it is preferable to include a preschool teacher in the study (if parents agree to their being contacted), this is not a prerequisite or an inclusion criterion (Note to File G003 25 May 2016; positive Ethics Committee vote 3 June 2016). Fifth, initially, an exclusion criterion was “current medication for ADHD or other psychotropic medication”. After realising that this exclusion criterion leads to an exclusion of severe cases with a current ADHD medication, this criterion was changed to “psychotropic medication of the child (except for ADHD medication)” (Note to File G003 25 May 2016, positive Ethics Committee vote 3 June 2016).

This table gives the inclusion and exclusion criteria of Version 3 of the Trial Protocol (V03, 14. October 2016) including all amendments G001-G005.

** The comorbid conditions which are defined as exclusion criteria are the same as those for the different trials within the ESCAlife consortium, including some diagnoses which are unlikely to appear at preschool age.

Patients are included if they meet the eligibility criteria (see Table 1). Parents (and, if applicable, preschool teachers) must give their informed consent and children their assent for study participation.

### Response criteria

The response criteria correspond to the child’s symptom inclusion criteria (see Table 1). Partial or non-responders have ADHD (DSM-5) assessed with the clinician-rated ADHD Checklist (DCL-ADHS) or ODD (DSM-5) assessed with the clinician-rated ODD Checklist (DCL-SSV) plus substantial ADHD symptoms (defined by a score of ≥ 0.7 on either of the two subscales [hyperactivity/impulsivity or inattention] of the DCL-ADHS). Full responders fulfil neither of these conditions and no longer have ADHD or ODD with substantial ADHD symptoms.

### Data handling

All legal requirements pertaining to the protection of personal data have been met. Upon enrolment, every participating child is assigned with a study-specific identification code. To ensure complete pseudonymisation, all study data from patients and their parents are stored under their assigned code. This is not shared, with the sole exception of transmission of contact details to members of the Cologne group providing the TASH intervention, following consent from participating parents. Only the PI and the study coordinators at each site have access to the patient identification list. The Clinical Trials Unit (CTU) Freiburg provides an electronic remote data entry system (RDE-LIGHT), in which information is entered by specifically trained personnel under the study code. To prevent unauthorised access to confidential participant information, built-in security features encrypt all data before transmission to and from the CTU. Users who enter data into the system are registered with the CTU and receive an individual ID and password to gain access to the system, in order to prevent unauthorised access to patient data. Data processing at the CTU is limited to authorised personnel who are familiar with the data handling procedures according to the study protocol.

### Interventions

#### Telephone-assisted self-help (TASH)

In step 1, all participants receive a 3-month behaviour therapy-oriented TASH intervention for parents of children with externalising behaviour problems. Additionally, if both the parents and the preschool teacher agree, preschool teachers also receive the intervention [40, 41]. The intervention is based on the *Therapy Programme for Children with Hyperactive and Oppositional Problem Behaviour* (*Therapieprogramm für Kinder mit hyperkinetischem und oppositionellem Problemverhalten* – *THOP* [42]) and the German self-help book *Wackelpeter & Trotzkopf* [43]. Both the parent and the preschool teacher programme consist of self-help booklets on externalising behaviour problems and behaviour modification techniques, which are sent to the participants through the post. Additionally, they receive telephone consultations with a therapist in advanced training for child and adolescent psychotherapy, who is supervised by senior supervisors. These consultations serve to support the parents and preschool teachers with the implementation of the interventions into their daily routines [40, 41].

Parent TASH programmes similar to that used in the present study have already been shown to reduce behaviour problems in preschool- and school-age children (e.g. [30, 32–34]). For the current study, the parent booklets were revised and adapted to address the specific needs of families of preschool children. The parents receive eight booklets and ten telephone consultations, each lasting for approximately 30 minutes. The TASH programme for the preschool teachers consists of four newly developed booklets and four telephone consultations of up to 60 minutes each. The appointments for the telephone consultations are set on an individual basis. The contents of the booklets are described in Table 2 and Additional file 1: Table S1.

**Table 2:** Overview of the telephone-assisted self-help (TASH) booklets for parents of preschool children

	Title	Content
1	Taking a close look at our problems	Defining individual problem behaviour and psychoeducation regarding coercive parent-child interactions
2	What is ADHD?	Psychoeducation on ADHD symptoms in preschoolers, associated problems, reasons for ADHD, the developmental course of ADHD, and treatment alternatives
3	Learning to like each other again	Encouragement of positive parent-child interactions by focusing on positive traits and positive experiences with the child and by actively creating more positive interactions and experiences with the child
4	Clear daily structures – gathering energy and implementing clear rules	Implementation of well-structured daily and weekly routines, strategies of parental stress management, and reflecting on and implementing family rules
5	Make effective requests and do not skimp on praise	Making effective requests, praise and positive consequences for following rules
6	The need for consequences	Appropriate negative consequences for breaking rules
7	If praise is not enough: reward systems	Implementation of reward symptoms
8	Learning how to play attentively	Helping the child to stay attentive when playing

For the preschool teachers, the interventions originally developed for the family environment have been adapted to the preschool environment. Moreover, the booklets for the preschool teachers cover information on improving environmental conditions, which might help the child to deal with his or her behaviour problems, and on constructive cooperation with parents (see Additional file 1: Table S1).

If children are full responders to the TASH intervention, two additional telephone consultations for the parents and, optionally, one additional telephone consultation for the preschool teacher are provided (Booster TASH).

### Parent management and preschool teacher training (PMPTT)

For children with no or only partial response to TASH, who are randomised to the PMPTT group, age-appropriate individually tailored behaviour therapy is provided in step 2. The 6-month PMPTT encompasses (1) parent management training, including parent-child interaction training, (2) preschool-teacher-focused interventions, including psychoeducation and behavioural interventions in the preschool, and (3) child-focused interventions (see Table 3). The primary goal of PMPTT is to reduce child problem behaviour and to enhance the parent-child and teacher-child relationship by improving parents’ skills and teachers’ educational skills. A total of 20 weekly sessions are provided. Parent- and teacher-focused interventions are based on the THOP [42] and on the PEP (*Prevention Programme for Externalising Problem Behaviour*; *Präventionsprogramm für Expansives Problemverhalten -* PEP [44]). Child-focused interventions are based on the *Therapy Programme for the Improvement of Organisational Skills, Concentration and Impulse Control in Children with ADHD* (*Therapieprogramm zur Steigerung von Organisationsfähigkeit, Konzentration und Impulskontrolle bei Kindern mit ADHS* – THOKI-ADHS [45]). THOP is the only German treatment programme for ADHD with established efficacy [42]. PEP has been extensively evaluated in several trials with preschool children with externalising problem behaviour [9]. The PEP programme was originally developed for the training of parents and teachers of preschool children in a group format. For ESCApreschool, PEP materials were adapted for use in an individual format. PMPTT is conducted by clinical therapists who are trained during a 2-day workshop (see section on Treatment integrity).

**Table 3:** Overview of the Parent Management Preschool Teacher Training (PMPTT) contents

Introductory therapy components (sessions 1–5)					
	Parents (+ child)		Child		Preschool teacher
P1	Getting to know each other and exploration of current externalising symptoms of the child	C1	Getting to know each other and exploration of the child	T1	Exploration of current externalising symptoms of the child in the preschool environment
P2	More specific exploration of the current ADHD symptoms of the child	C2	Clinical observation of a structured play situation	T2	Psychoeducation on ADHD
P3	Psychoeducation on ADHD				
Basic therapy components (sessions 6–10)					
	Parents (+ child)		Preschool teacher		
P4	Focusing on positive experiences with the child	T3	Encouragement of positive teacher-child interactions		
P5	Implementation of clear rules (optionally together with the child)				
P6	Development of effective requests				
P7	Social reinforcement and positive consequences of following rules				
P8	Appropriate negative consequences of breaking rules				
Intensification components (sessions 11–20)					
P9	Social reinforcement of non-disturbing behaviour	T4	Helping the child to stay attentive when playing		
P10	Development of positive play interactions	T5	Implementation of clear daily structures		
P11	Development of token systems	T6	Implementation of clear individual rules and group rules		
P12	Development of response cost systems	T7	Development of effective requests		
P13	Time-out	T8	Appropriate positive or negative consequences of following or breaking rules		
P14	Strategies of parental stress management	T9	Development of token systems or response cost systems		
P15	Fostering the child’s strengths and interests and channelling his or her energy				
P16	Helping the child to stay attentive when playing (participant: child, optionally together with parent)				
P17	Management of behaviour problems in public				
P18	Consultation regarding non-externalising / emotional problems of the child				

If possible, introductory therapy components and basic therapy components should be applied in all families. Intensification components are used depending on the individual needs of the families

### Treatment as usual (TAU)

The other group of partial or non-responders to TASH receives TAU in step 2, that is, a typical, mostly guideline-based ADHD preschool intervention. TAU is usually conducted by the participating centres but may also be performed by local cooperating institutions (e.g. child guidance centres, ergo−/occupational therapy, child and adolescent psychiatrists, psychotherapists). In the latter case, members of the cooperating institutions are asked to provide information about their treatment. To achieve a minimum of conformity, at least four patient contacts within the treatment period of 6 months are recommended.

Starting or optimising an additional ADHD pharmacotherapy (according to the clinical decision of the treating physician) is permitted in step 2 in the TAU condition as well as in the PMPTT condition, but has to be documented.

### Treatment integrity

Treatment integrity is established through qualification standards for therapists (therapists have completed a university degree qualifying for training to become a licensed child and adolescent therapist, and are currently in training for psychotherapy with children and clinical expertise in the treatment of ADHD), study-specific therapist training, the use of the manualised treatment programmes and the use of protocol sheets for treatment documentation. TASH and PMPTT treatments are supervised by senior supervisors to check for adherence to the manual and study procedures. PMPTT therapists participate in three supervision sessions per patient. These sessions are scheduled after therapy sessions 5, 10 and 15, and are conducted either face to face or by telephone. For each patient, at least two video sequences are discussed with the supervisor. The TASH consultations are recorded in audio files and supervised regularly.

### Informants

ESCApreschool collects information from different perspectives and informants: unblinded clinician (e.g. therapist or TASH counsellor), blinded clinician, the participating parent, the other parent/partner of participating parent, and, optionally, the preschool teacher.

The blinded clinicians conduct the clinical interviews with the parents, and are blind to the study condition. However, in order to minimise heterogeneity in ratings caused by different assessors, the same clinician should perform the interviews with a family at the different assessment time points. Therefore, the blinded clinicians are not blind to the assessment time point.

Additionally, to control for inter-rater reliability, the ratings of the parent interviews are audio- or videotaped. A clinician who is blind to both the treatment condition and the assessment time point rates a random selection of these interviews. The parent is the biological parent or guardian of the child, who is involved in the treatment (he or she is therefore not blind to the treatment condition). If possible, ratings by the child’s preschool teacher are obtained.

### Measures

#### Main assessment time points

**Fig. 2:**
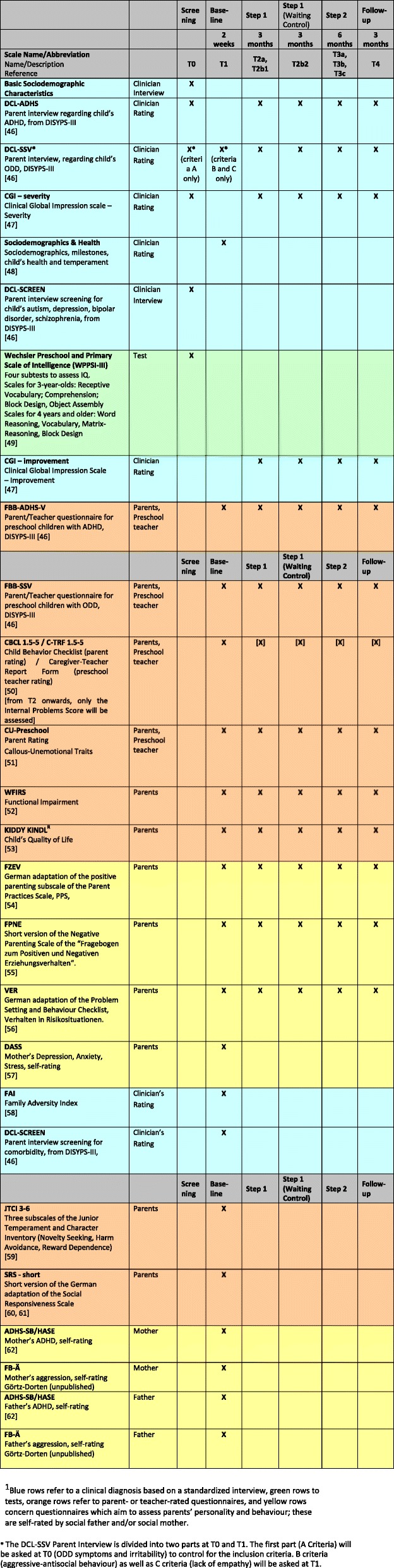
Overview of measures and assessment time points

Unless otherwise stated, the primary and secondary outcome measures are assessed at all four main assessment time points (T0/T1, T2, T3, T4). Figure 2 gives an overview of the measures assessed at the different time points.

Additional file 2: Figure S1 presents the time schedule in greater detail, with an overview of outcome measures, predictors and eligibility criteria.

### Primary and secondary outcome measures

#### Primary outcome measures

The primary outcome is the change in the combined ADHD and ODD symptom score. This combined symptom score is derived from the blinded clinician-rated ADHD and DBD Symptom Checklists based on a parent interview (DCL-ADHS + SSV Parent [46]). The DCL-ADHS and the DCL-SSV assess symptoms of ADHD or ODD and conduct disorder, respectively, according to DSM-5 and ICD-10 criteria. The DCL-ADHS consists of 18 items assessing ADHD symptoms and five items assessing functioning and psychological strain. The items on ADHD symptoms can be aggregated into two scales, *inattention* (nine items) and *hyperactivity/impulsivity* (nine items). The DCL-SSV comprises 28 items belonging to four subscales: *ODD* (eight items), *aggressive-dissocial problem behaviour* (seven items), *limited prosocial emotionality* (11 items), and *disruptive mood disorder* (five items, three of which are also part of the *ODD* scale). For ESCApreschool, we excluded the scale assessing disruptive mood disorder, as the associated symptoms are uncommon in preschool children. Moreover, the DCL-SSV includes five items on *functioning* and *psychological strain*. All items are rated on a 4-point Likert-type scale ranging from 0 to 3, with higher scores indicating higher symptom severity. Scale scores are computed by averaging the associated item scores. The DCL-ADHS and the DCL-SSV subscales and total scores show satisfactory internal consistency (Cronbach’s α > .68 [46, 63].

We use a combined score of ADHD and ODD, since the two conditions are highly correlated in preschool children with ADHD, and the reduction of symptoms of ADHD and ODD is usually the main objective of treatments in this age group.

### Secondary outcome measures

The secondary outcome measures assess (1) ADHD and DBD symptoms as rated by parents and teachers, (2) psychosocial impairment, (3) comorbid symptoms and comorbid mental disorders, (4) quality of life of the child as perceived by parents, (5) social reactivity, and (6) parenting behaviour. All instruments used to assess symptoms and impairment are recommended in diagnostic guidelines for ADHD [12] and are employed in clinical practice. All secondary outcome measures have been validated both in English and in German and have been used in previous trials of pharmacological and psychotherapeutic interventions as well as in prevention studies. They have been widely used in children, and especially in preschool children with ADHD. All instruments provide population-based norms and therefore enable the calculation of age- and gender-adjusted normalisation rates.

### ADHD and DBD symptoms

Parents and preschool teachers rate the symptom severity of ADHD and DBD on the *ADHD Parent and Teacher Rating Scale for Preschool Children* (FBB-ADHS-V; German: *Fremdbeurteilungsbogen für Vorschulkinder mit Aufmerksamkeitsdefizit−/Hyperaktivitätsstörungen* [46]) and on the *DBD Parent and Teacher Rating Scale* (FBB-SSV; German: *Fremdbeurteilungsbogen für Störungen des Sozialverhaltens*). The scales capture symptoms of ADHD or DBD, respectively, according to ICD-10 and DSM-5. The FBB-ADHS-V consists of 19 items belonging to the subscales *inattention* and *hyperactivity/impulsivity*. In the version for younger children (up to the age of 11), the FBB-SSV comprises 27 items, which can be aggregated into four subscales: *ODD*, *aggressive-dissocial problem behaviour*, *limited prosocial emotionality*, and *disruptive mood disorder*. All items are rated on a 4-point Likert-type scale ranging from 0 to 3, with higher scores indicating higher symptom severity. Scale scores are computed by averaging the associated item scores. The subscales and the total score of both the FBB-ADHS and the FBB-SSV have demonstrated reliability and factorial validity.

### Clinical global impression and functional impairment

The clinician-rated *Clinical Global Impression Scale* (CGI) is administered using a short clinical interview [47]. It is a widely used outcome parameter in clinical trials, measuring disease severity (CGI-severity, CGI-S) as well as general improvement during treatment (CGI-improvement, CGI-I). Both the CGI-S and the CGI-I are rated on a 7-point scale, with higher scores indicating greater severity or improvement, respectively. The CGI shows a good inter-rater reliability (.65–.92 [64];) and an intra-class correlation coefficient of .91 [65].

A German version of the parent-rated *Weiss Functional Impairment Rating Scale*, which was modified and adapted for use in preschool-age children, is applied to measure functional impairment [66, 67]. The modified German version consists of 40 items, which are rated on a 4-point Likert-type scale ranging from 0 to 3. Higher scores indicate greater impairment. The subscales and total score of the original version and of the modified German version have shown internal consistency (α > .80) and factorial validity [66–68]. Moreover, the original version has demonstrated test-retest reliability, convergent validity, and responsiveness to change [68].

### Comorbid internalising and externalising symptoms

The German version of the parent-rated *Child Behavior Checklist 1½-5* (CBCL 1½-5 [69];; original English version: [70]) and the preschool teacher-rated *Caregiver-Teacher Report Form 1½-5* (C-TRF 1½-5; Arbeitsgruppe Deutsche Child Behavior Checklist, 2002b; original English version: [70]) are questionnaires to assess behavioural problems, emotional problems and somatic complaints of toddlers and preschool children aged 1½ to 5 years. Both scales comprise 99 items (83 overlapping items) rated on a 3-point scale ranging from 0 to 2. The items can be aggregated into several syndrome scales as well as into three superordinate scales: *internalizing problems*, *externalizing problems*, and *total problems*. These superordinate scales are considered in ESCApreschool. For both the CBCL and the C-TRF, these scales have demonstrated satisfactory internal consistencies (α > .80) in German samples [69]. Moreover, analyses in German samples have provided evidence for the construct validity of the CBCL as well as limited evidence for the factorial validity of the CBCL *internalizing problems* and *externalizing problems* scales [69].

### Quality of life

In evaluating health care with respect to prevention and treatment, quality of life has emerged as an important concept. We use the Kiddy-KINDL [53, 71, 72] to assess subjective generic health-related quality of life in preschool children. Results on the reliability of this scale vary: In a sample of preschoolers aged 4–6 years, Cronbach’s alpha for the total score varied from .66 to .70 depending on age and gender [72]. However, in a larger sample of 3–6-year-old children, a Cronbach’s alpha of .82 was reported [73].

### Callous-unemotional traits and social responsiveness

Callous-unemotional traits are assessed with five items of the *prosocial behaviour* scale and the *peer problems* scale of the *Strengths and Difficulties Questionnaire* (SDQ [74]) and three items of the callous-unemotional dimension of the *Antisocial Process Screening Device* (APSD-CU [75], which were combined into a joint measure. The items are rated on a 3-point scale ranging from 0 to 2. Pasalich et al. [51] also combined items of the SDQ and the APSD, but used more items. They found satisfactory internal consistencies across mother, father and child ratings of their measure (range of α = 0.69–0.87).

Social responsiveness is assessed using a shortened 16-item version of the *Social Responsiveness Scale* (SRS-short [76]). The items of this questionnaire are rated on a 4-point scale ranging from 0 to 3. The long version of the SRS shows high internal consistency (α = 0.91–0.97) as well as satisfactory test-retest reliability, inter-rater reliability, convergent validity and discriminative validity [60].

### Parenting behaviour

Positive parenting behaviour is assessed using a German questionnaire covering positive parenting skills (*Fragen zum Erziehungsverhalten*, FZEV; Naumann, Kuschel et al., 2007). This questionnaire comprises 13 items rated on a 4-point scale ranging from 0 to 3, with higher scores indicating more positive parenting behaviour. The scale shows satisfactory internal consistency (mother ratings: α = 0.85; father ratings: α = 0.87 [77]). For the assessment of negative parenting behaviour, we apply the respective 13-item scale of the *Questionnaire on Positive and Negative Parenting Behaviour* (German: *Fragebogen zum positiven und negativen Erziehungsverhalten*, FPNE [55]). The items of this scale are rated on a 4-point scale ranging from 1 to 4, with higher scores indicating more negative parenting behaviour. The scale is internally consistent (α = 0.78).

Additionally, to assess parents’ perceived sense of competency concerning challenging parenting situations, we employ a modified German Version of the *Problem Setting and Behaviour Checklist* (German: *Verhalten in Risikosituationen*, VER [77]). The 27 items of this questionnaire are rated on a 4-point scale ranging from 1 to 4, with higher scores indicating a stronger sense of competency. The scale has demonstrated high internal consistency (mother ratings: α = 0.92, father ratings: α = 0.94 [77]).

### Treatment satisfaction

For the assessment of *satisfaction* with the treatment, treatment-specific parent satisfaction questions were developed (e.g. for the assessment of satisfaction with TASH). These are assessed as part of the clinical interview at T2 and T3.

### Feasibility and adherence measures

Besides the outcome measures used at all of the main assessment time points (T1 to T4), the following measures are used to assess feasibility and adherence:

1. *Clinical Feasibility Rating Scale* (newly developed) to rate the feasibility of the TASH and PMPTT interventions (at T2 and T3);
2. *Clinical Adherence Rating Scale* (newly developed) to assess the adherence of the patient, the parents and preschool teachers during the interventions (at T2 and T3).

### Assessment of potential moderators of treatment response

The following potential moderators of treatment response are analysed: (1) age, (2) gender, (3) socioeconomic status, (4) ADHD symptom severity, (5) comorbid symptoms, (6) intelligence, (7) parental depression, anxiety and stress (*Depression, Anxiety and Stress Scale*, DASS; Cronbach’s α = 0.89–.96, test-retest reliability *r* = 0.71–.81, validity [57, 78]), and (8) parental ADHD (*ADHD Self-Rating Scale*, German: *ADHS-Selbstbeurteilungsskala*, ADHS-SB; test-retest reliability *r* = 0.78–.89; Cronbach’s α = 0.72–.9, validity [62, 79]).

### Psychometric data

The following variables are assessed using a clinical interview with the parent during the diagnostic assessment at T0 and T1 and considered as possible predictors of treatment outcome: sociodemographic data of the child and the parent (e.g. child age, educational level of the parent or guardian), data on *early child development* (six items), temperament (13 items; *Junior Temperament and Character Inventory* [JTCI] 3–6 [59]), irritability (seven items; *Affective Reactivity Index* [ARI-Parent] [80]), and *life events* (14 items). Additionally, we employ the German version of the *Family Adversity Index* (FAI), adopted from the German *Mannheimer Elterninterview* [81]; original English version: [82, 83]). To measure parental aggression, we use the anger control scale of the German *Elternfragebogen zum Umgang mit Ärger* (FB-Ä) [Götz-Dorten, A. (unpublished) 2013; Department of Child and Adolescent Psychiatry, Psychosomatics and Psychotherapy, University of Cologne], which is a modified version of the 12-item form of the Aggression Questionnaire [84, 85]. The clinical checklist *Diagnose-Checkliste zum Screening psychischer Störungen* (DCL-SCREEN; taken from the DISYPS-III [46]) is used to assess comorbid symptoms of depression (seven items), anxiety (ten items), autism spectrum disorder (four items), other neurodevelopmental disorders (six items), obsessive-compulsive disorder (two items) and tic disorders (one item). Based on a modified questionnaire by Piacentini et al. [86], the therapists additionally report their *expectation of treatment benefit* (three items) for a family. Moreover, the participating parents provide information about their own *treatment expectations*.

Furthermore, after every therapy session of step 1 and step 2, the therapist rates the *treatment integrity* (13 self-developed items), the *treatment adherence* of the client (ten items; eight items for TASH only), and current ADHD symptoms of the child (four items; shortened version of the German *ADHD Questionnaire* [87].

At the beginning of the study (T0/T1), children complete four subtests of the *Wechsler Preschool and Primary Scale of Intelligence* (WPPSI-III) to assess their IQ [49]. The 3-year-old children work on the scales *Receptive Vocabulary*, *Comprehension*, *Block Design* and *Object Assembly*, while the 4–6-year-olds complete the subtests *Word Reasoning*, *Vocabulary*, *Matrix-Reasoning* and *Block Design*.

### Biological data

Transcranial sonography (TCS) is used to assess biological predictors of treatment response, especially in younger children. This is part of the transversal project ESCAbrain, which assesses biological data in all ESCAlife trials (see also [37–39]). Using TCS, the size of the echogenic region of the substantia nigra is assessed. In children, ADHD-associated hyperechogenicity of the substantia nigra has consistently been reported and has previously been identified as a potential biological marker of ADHD [88, 89]. TCS is a non-invasive method for the visualization of deep brain structures, such as the substantia nigra, through the intact skull. Ultrasound waves are reflected depending on tissue composition, resulting in different echogenicity of nuclei and ventricular system [38]. The method has no harmful side effects. Of particular interest is the mesencephalic scanning plane, including brainstem, substantia nigra and raphe nuclei. In terms of clinical implications, TCS can aid differential diagnosis (e.g. in movement disorders [90]) and has shown promise in predicting treatment response in psychiatric disorders in adult patients. As yet, no study has explored whether TCS can predict the effectiveness of non-pharmacological interventions. TCS is optional for the patients. As a well-tolerated investigation, it offers fast, non-invasive, targeted imaging in this age group in which magnetic resonance imaging (MRI) is not feasible or practical.

Furthermore, saliva samples are collected before step 1 (T0/T1) and after step 2 treatment (T3) to determine predictive genetic and epigenetic patterns. These saliva samples are collected according to standard protocol of the ESCAmark subproject, which coordinates biosampling across the ESCAlife consortium. Samples are stored at − 80 °C and will be analysed after recruitment closure of all RCTs within ESCAlife. Samples will be used according to the ethics vote and data management plan of ESCAmark that will be published separately.

### Randomisation procedure

Central randomisation with a 1:1 treatment ratio, analogous to the other ESCA trials [37–39], is performed by the CTU at the University Medical Centre Freiburg via fax, using block randomisation with variable block length to ensure concealment of randomisation. Randomisation is stratified by centre. The randomisation request form contains the study-specific patient identification number, year of birth and the confirmation of ADHD above the cut-off. The CTU reviews the patient’s details on the randomisation fax and performs the randomisation if the data on the fax are appropriate and complete.

### Quality assurance and monitoring

The monitoring is performed by the clinical research associates (CRAs) of the CTU. Adapted monitoring is accomplished according to Good Clinical Practice (ICH-GCP E6) and standard operating procedures (SOP). This verifies that patients’ rights and well-being are protected, that reported trial data are accurate, complete and verifiable from source documents, and that the trial is conducted in compliance with the currently approved protocol/amendment, with GCP and with the applicable regulatory requirements to ensure safety and integrity of clinical trial data. In this trial, all trial-specific monitoring procedures, monitoring visit frequency and the extent of source data verification (SDV) are predefined in a specific monitoring manual.

The investigator accepts monitoring visits before, during and after the clinical trial. Prior to the trial, a pre-trial telephone consultation and a site initiation visit at each site are conducted in order to train and introduce the investigators and their staff to the trial protocol, essential documents and related trial-specific procedures, ICH-GCP and national/local regulatory requirements.

During the trial, the monitor visits the site regularly depending on the recruitment rate and quality of data. During these on-site visits, the monitor verifies that the trial is being conducted according to the trial protocol, trial-specific procedures, ICH-GCP and national/local regulatory requirements. Moreover, the monitor checks that signed informed consent has been provided, and verifies the eligibility of patients, completeness of primary endpoint questionnaires, treatment compliance, and documentation. The monitor also performs source data verification to ensure that clinical trial data are recorded and documented in the source data and that case report forms (CRFs) are complete and accurate. In the case of data quality problems or a high number of protocol violations at individual sites, the extent of source data verification and frequency of monitor visits is adapted accordingly.

The investigator must maintain source documents for each patient in the trial, consisting of case and visit notes (hospital or clinic medical records) containing demographic and medical information, laboratory data, and the results of any other tests or assessments. All information recorded on CRFs must be traceable to source documents in the patient’s file. The investigator must also keep the original signed informed consent form (a signed copy is given to the patient).

The investigator must give the monitor access to all relevant source documents to confirm their consistency with the CRF entries.

An independent Data Monitoring Committee (DMC), composed of Prof. Dr. H. J. Freyberger, Prof. Dr. A. Rothenberger and Prof. Dr. J. Schmitt, advises the trial sponsor on patient safety and measures to ensure the credibility and integrity of the ongoing trial.

### Stopping rules

Inclusion in the study is not possible without written informed consent of parents/guardians. If a preschool child requires inpatient treatment or needs a different kind of treatment for health reasons according to the judgment of the attending physician, the child will be excluded from the study. Study exclusion will also occur if any other factors arise which affect the child’s well-being. The Ethics Committee will be informed immediately in the case of severe events during the conduct of the trial. Global stopping rules for the trial or closing of a centre include the emergence of data leading to a revision of the risk-benefit ratio, ongoing failure of recruitment, or repeated violations of standard GCP rules or of the study protocol. For a decision on the termination of the trial or on closing a participating centre, agreement between the study coordinator, PIs, site investigators, DMC members, the responsible Ethics Committee and the CTU Freiburg is intended.

### Sample size and power calculations

The whole stepped care design is primarily powered for the two RCTs in step 1 and step 2. Based on the results of previous studies, an effect size of *d* = 0.5 is expected for the RCT in step 1 (TASH compared to a waitlist control group). Kierfeld et al. [30] found a moderate to large effect (*d* = 0.79) of a TASH intervention compared to a waitlist control group on parent-rated ADHD symptoms. Both the intervention and the outcome measure were similar to those used in this current trial. Effect sizes of about SMD = 0.75 were found for parent management training interventions, mainly compared to waiting groups in children with disruptive behaviour problems, using unblinded parent ratings [18]. However, reported effects sizes are smaller when blinded ratings are applied [23]. Therefore, we expect an effect size of *d* = 0.5 for the primary outcome for the randomisation comparing PMPTT with TAU in step 2.

The calculation of the sample size (software: STPLAN Version 4.3) is based on the primary endpoint of step 2 (ADHD/ODD change score from T2 to T3). Using a two-sided *t* test with a power of 80% at a significance level of 5%, 64 patients with non-missing data per group are required to detect a difference when the true effect size is *d* = 0.5. To account for the possibility that some patients (10%) will have incomplete data at T3, in total, 144 partial or non-responders should be randomised at step 2. We assume that about 20% (*n* = 36) will show a full response after step 1 [30] and that a group of 10% will have dropped out from T1 to T2. Therefore, 180/0.9 = 200 patients should be randomised at T1 (step 1). Given 180 patients with complete data and a presumed true effect size of *d* = 0.5, the power to detect a difference is 92%. We assume that about 300 patients will need to be screened for study participation.

### Statistical analyses

Before the inclusion of the first patient, a detailed statistical analysis plan (SAP) was prepared. This will be completed during the ‘blind review’ of the data, at the latest. If the SAP contains any changes to the analyses outlined in the trial protocol, they will be marked as such, and reasons for amendments will be given.

All statistical programming for analysis will be performed with the Statistical Analysis System (SAS Institute, Cary, NC, USA).

### Definition of populations included in the analyses

The primary analysis will be conducted according to the intention-to-treat (ITT) principle. This means that the patients will be analysed in the treatment arms to which they were randomised, irrespective of whether they refused or discontinued the treatment or whether other protocol violations become apparent.

The per-protocol (PP) population is a subset of the full analysis set (FAS) and is defined as the group of patients who had no major protocol violations, received a predefined minimum dose of the treatment and underwent the examinations required for the assessment of the endpoints at relevant, predefined times. The analysis of the PP population will be performed for the purpose of a sensitivity analysis.

Safety analyses will be performed in the safety population. Patients in the safety population are analysed as belonging to the treatment arm defined by treatment received. Patients are included in the respective treatment arm if treatment was started/if they received at least one dose of trial treatment.

### Patient demographics/other baseline characteristics

Demographic and other baseline data (including disease characteristics) will be summarised descriptively using all documented patients. Continuous data will be summarised by arithmetic mean, standard deviation, minimum, 25% quantile, median, 75% quantile, maximum, and the number of complete and missing observations. If appropriate, continuous variables can also be presented in categories. Categorical data are summarised by the total number of patients in each category and the number of missing values. Relative frequencies are displayed as valid percentage (number of patients divided by the number of patients with non-missing values).

### Analysis of primary endpoint

The primary statistical analyses of steps 1 and 2 will be by ITT, that is, all randomised patients will be analysed according to their allocated arm. Changes in the DCL ADHD + ODD (DBD) Parent scores between T1-baseline and T2 (after TASH/waiting) or T2 (after TASH) and T3, respectively, will be evaluated in separate mixed-effects models for repeated measures (MMRM). The MMRMs will include fixed categorical effects of treatment, centre, visit and treatment-by-visit interaction, and continuous, fixed covariates of baseline and baseline-by visit interaction. Further covariates predictive of missingness will be included based on a pre-specified selection strategy, to correct for potential bias arising from missing data.

Unstructured covariance matrices will be used to model within-patient correlations. The primary treatment comparisons of the change scores at T2 and T3 will be based on least-squares means with two-sided 95% confidence intervals without correction for multiple testing. Other possibly relevant covariates may be considered as well. Subgroup analyses will be conducted in an exploratory manner by including interaction terms in the MMRMs. These will focus on the analyses of patients’ and parents’ comorbidity. In addition, gender effects will be investigated as prognostic and predictive factors. Exploratory within-subjects comparisons (change in step 1 compared to change in step 2) will also be also carried out in MMRMs. Secondary efficacy endpoints derived from other scale scores will be analysed in the same manner, i.e. with the same type of linear model. Follow-up of full responders after step 1 will be evaluated descriptively. No interim analysis for efficacy will be performed.

Safety/tolerability analyses will be carried out in all patients for whom one of the randomised treatments was started, according to treatment received.

### Analysis of secondary endpoints

Secondary endpoints will be analysed descriptively in a similar fashion to the primary outcome. Scores will be calculated according to the respective manuals.

The analysis of the change between T1-baseline and T2 (after TASH/waiting) and T3 (after TAU/PMPTT) comprises the analysis of the primary endpoint for continuous measurements (DCL-ADHS, DCL-SSV, FBB-ADHS, FBB- SSV, CU-Preschool, CBCL/1,5–5, CTRF, WFIRS, KIDDY KINDL, FZEV, FPNE, VER). In addition, for DCL-ADHS and the measurements listed above, change between T2 and T3 in patients without ADHD/ODD (at T2) will be evaluated. Treatment effects will be calculated with two-sided 95% confidence intervals.

The within-patient changes between T2 and T3 in patients without ADHD/ODD (at T2) in continuous endpoints will be analysed using linear regression adjusted for the baseline measurement and study centre.

The difference in the CGI between T1 and T2 and between T2 and T3 in the randomised steps will be analysed using the Mann-Whitney *U* test. The within-patient difference in the CGI will be analysed using the Wilcoxon signed-rank test.

Possible predictors of the DCL-ADHD score will be analysed using linear regression.

### Legal and ethical foundation

Before trial start, all relevant documents were submitted to the local Ethics Committee responsible for the respective participating centres. The primary vote on the study was obtained from the Ethics Committee of the Medical Faculty of the Philipps University of Marburg. For changes to the trial protocol that are formal in nature or include relevant changes for participants, the ethics committees have to vote anew.

## Discussion

ESCApreschool (investigating 3–6-year-old preschool children with ADHD) is one part of the multicentre study ESCAlife, which examines clinical care for children, adolescents and adults with ADHD to optimize evidence-based personalised stepped care approaches across different age groups (see also [37–39]).

Early onset, high prevalence and persistence, and developmental comorbidity make ADHD a psychosocially impairing and cost-intensive mental disorder. Despite continuous treatment research, there is still a substantial need to optimise individualised treatment strategies in order to improve outcomes and reduce the economic burden. By covering the full spectrum of ADHD at all ages, the consortium will be able to make significant recommendations for improving ADHD treatment in routine clinical care.

Clinical guidelines recommend an adaptive treatment and a stepped care approach for the treatment of ADHD/ODD [12]. However, this approach has not yet been empirically validated. The main goal of ESCApreschool is therefore to assess the efficacy of a stepped care approach in children with ADHD/ODD aged 3–6 years and to identify predictors as well as moderators of treatment outcome. The design combines two RCTs. The first aims to analyse the efficacy of the low-threshold TASH intervention by means of a randomised waiting control design and to identify predictors of response. Partial and non-responders to TASH take part in a second RCT, which compares the effects of an intense behaviour therapy addressing parents, children and preschool teachers with TAU. Thus, the design allows for the evaluation of the additional effects of behaviour therapy and TAU in preschool children with ADHD/ODD who did not sufficiently respond to the low-threshold intervention in step 1 of the study.

The results will improve future guidelines on the treatment of preschool-age children with ADHD and/or ODD. Moreover, the findings may also be used to develop usable, potentially more cost-effective, individualised stepped care pathways for young children with ADHD/ODD. The evaluation of predictors of treatment response will help to identify indications for specific treatments during the therapy process. Resource-intensive therapeutic interventions can then be specifically directed at individuals who will probably not respond to or benefit from low-threshold treatment. This would help to distribute resources efficiently and improve treatment for preschool psychiatric patients. Such a targeted, resource-friendly use of interventions will help children with ADHD, their families, preschool teachers, peers, the health care system and society as a whole.

### Trial status

The trial was registered at the German Clinical Trials Register (DRKS) as a Current Controlled Trial under DRKS00008971 on 1 October 2015. This manuscript is based on protocol version 3 (14 October 2016). Recruitment started January 2016 with the first patient enrolled on 29. June 2016 (first patient in). The milestone of 75% (150 patients) was reached in June 2019. Recruitment for this trial is ongoing. Recruitment will be completed in approximately December 2019.

## Supplementary information

**Additional file 1: Table S1.** Overview of the telephone-assisted self-help (TASH) booklets for preschool teachers.

**Additional file 2: Figure S1.** Time schedule with an overview of outcome measures, predictors and eligibility criteria.

**Additional file 3.** SPIRIT 2013 Checklist: Recommended items to address in a clinical trial protocol and related documents.

## Data Availability

Not applicable.

## Abbreviations

ADHD: Attention-Deficit/Hyperactivity Disorder
AKiP: School of Child and Adolescent Cognitive Behaviour Therapy [Ausbildungsinstitut für Kinder- und Jugendlichenpsychotherapie]
Booster SH: Booster Self-Help
CAP: Child and Adolescent Psychiatry
CBCL 1½-5: Child Behaviour Checklist for Ages 1½-5
CGI: Clinical Global Impression Scale
CRF: Case report form
C-TRF: Caregiver and Teacher Report Form of the Child Behaviour Checklist
CTU: Clinical Trials Unit
CUpreschool: Parent Rating Callous-Unemotional Traits (preschool age)
DASS: Depression Anxiety Stress Scales
DBD: Disruptive behaviour disorder
DCL-ADHS: Clinical ADHD Checklist [*Diagnose-Checkliste für Aufmerksamkeitsdefizit−/Hyperaktivitätsstörungen*]
DCL-SCREEN: Clinical Checklist for Screening Mental Disorders [*Diagnose-Checkliste zum Screening psychischer Störungen]*
DCL-SSV: [*Diagnose-Checkliste für Störungen des Sozialverhaltens*]
DGKJP: German Association for Child and Adolescent Psychiatry, Psychosomatics and Psychotherapy [*Deutsche Gesellschaft für Kinder- und Jugendpsychiatrie, Psychosomatik und Psychotherapie*]
DISYPS-III: Diagnostic System for Mental Disorders in Children and Adolescents Version III [*Diagnostiksystem für psychische Störungen nach ICD10 und DSM-5 für Kinder und Jugendliche-III*]
DMC: Data Monitoring Committee
DRKS: German Clinical Trials Register [*Deutsches Register Klinischer Studien*]
DSM-5: Diagnostic and Statistical Manual of Mental Disorders, Fifth Edition, Classification and Diagnostic Tool for Mental Disorders issued by the American Psychiatric Association
ESCAadol: Evidence-based stepped care of ADHD in Adolescents (aged 12–17 years)
ESCAlate: Evidence-based stepped care of ADHD in Adults (aged 16–45 years)
ESCAlife: Evidence-based stepped care of ADHD along the Lifespan
ESCApreschool: Evidence-based stepped care of ADHD in preschool children (aged 3–6 years)
ESCAschool: Evidence-based stepped care of ADHD in school children (aged 6–12 years)
FAI: Family Adversity Index
FAS: Full Analysis Set
FBB-ADHS: External Rating Questionnaire for ADHD [*Fremdbeurteilungsbogen für Aufmerksamkeitsdefizit/Hyperaktivitätsstörungen*]
FBB-SSV: Self-Assessment Questionnaire for ADHD [*Fremdbeurteilungsbogen für Störungen des Sozialverhaltens*]
FPNE: Questionnaire on Positive and Negative Parenting Behaviour [*Fragebogen zum positiven und negativen Erziehungsverhalten*]
FZEV: Questionnaire on Parenting Behaviour [*Fragen zum Erziehungsverhalten*]
ICD-10: International Classification of Diseases, Tenth Edition
ICH-GCP: Good Clinical Practice
ID: Identifier
IQ: Intelligence quotient
ITT: Intention-to-treat
JTCI: Junior Temperament and Character Inventory
Kiddy-KINDL: Instrument for assessing subjective generic health-related quality of life in preschoolers
MMRM: Mixed-effects model for repeated measures
MRI: Magnetic resonance imaging
ODD: Oppositional defiant disorder
PEP: Prevention Programme for Externalising Problem Behaviour [*Präventionsprogramm für Expansives Problemverhalten*]
PI: Principal Investigator
PMPTT: Parent Management and Preschool Teacher Training
pp.: per protocol
RCT: Randomized controlled trial
SAP: Statistical Analysis Plan
SDQ: Strengths and Difficulties Questionnaire
SDV: Source Data Verification
SMD: Standardised Mean Difference
SRS-short: Social Responsiveness Scale, short version
T0: Screening at the beginning of the study (to assess inclusion and exclusion criteria)
T0/T1: Indicates overlap in diagnostic procedure of T0 and T1 assessment
T1: Assessment at baseline before step 1
T2: Assessment at the end of step 1 and before the beginning of the step 2 intervention
T3: Assessment at the end of the step 2 intervention
T4: Follow-up assessment 3 months after the end of the step 2 intervention
TASH: Telephone-Assisted Self-Help for parents and preschool teachers
TAU: Treatment as usual
TCS: Transcranial sonography
THOP: Treatment Programme for Children with Externalising Disorders [*Therapieprogramm für Kinder mit hyperaktivem und oppositionellem Problemverhalten*]
VER: Questionnaire assessing behaviour in risk situations [*Verhalten in Risikosituationen*]
WFIRS: Weiss Functional Impairment Rating Scale – Parent Report
WPPSI-III: Wechsler Preschool and Primary Scale of Intelligence, third edition
