# Supplementary material for: Individualised stepwise adaptive treatment for 3–6-year-old preschool children impaired by attention-deficit/hyperactivity disorder (ESCApreschool): study protocol of an adaptive intervention study including two randomised controlled trials within the consortium ESCAlife
Source: Trials. 2020 Jan 9;21:56. doi: 10.1186/s13063-019-3872-8 (PMC6953462; doi:10.1186/s13063-019-3872-8)
Supplement: Supplementary file 1 — Additional file 1: Table S1. Overview of the telephone-assisted self-help (TASH) booklets for preschool teachers. [file 13063_2019_3872_MOESM1_ESM.docx]

**Additional file 1: Table S1**. Overview of the telephone-assisted self-help (TASH) booklets for preschool teachers

|  | Title | Content |
| --- | --- | --- |
| 1 | Taking a close look at our problems | - Psychoeducation on ADHD symptoms in preschoolers, associated problems, reasons for ADHD, the developmental course of ADHD, and treatment alternatives - Defining individual problem behaviour and psychoeducation regarding coercive teacher-child interactions |
| 2 | Building a stable foundation | - Reflecting on environmental conditions at preschool - Encouragement of positive teacher-child interactions by focusing on positive traits and positive experiences with the child and by actively creating more positive interactions and experiences with the child - Helping the child to stay attentive when playing - Cooperation with parents |
| 3 | Implement clear daily structures and rules and make effective requests | - Implementation of well-structured daily and weekly routines - Reflecting on and implementing rules - Making effective requests |
| 4 | Do not skimp on praise and stay consistent | - Praise and positive consequences for following rules - Appropriate negative consequences for breaking rules - Implementation of reward systems |
